# Impact of dietary regime on the metabolomic profile of bovine buttermilk and whole milk powder

**DOI:** 10.1007/s11306-024-02157-4

**Published:** 2024-08-03

**Authors:** Claire Connolly, Mark Timlin, Sean A. Hogan, Eoin G. Murphy, Tom F. O’Callaghan, André Brodkorb, Deirdre Hennessy, Ellen Fitzpartick, Michael O’Donavan, Kieran McCarthy, John P. Murphy, Xiaofei Yin, Lorraine Brennan

**Affiliations:** 1 UCD School of Agriculture and Food Science, Institute of Food and Health, UCD, Belfield, Dublin 4, D04 V1W8 Ireland; 2 UCD Conway Institute of Biomolecular and Biomedical Research, University College Dublin, Dublin, D04 V1W8 Ireland; 3 Food for Health Ireland, UCD, Belfield, Dublin 4, D04 V1W8 Ireland; 4 Teagasc, Food Research Centre, Moorepark, Fermoy, P61 C996 Co. Cork Ireland; 5 Teagasc, Animal and Grassland Research and Innovation Centre, Moorepark, Fermoy, P61 P302 Co. Cork Ireland; 6 Food for Health Ireland, Teagasc Food Research Centre, Moorepark, Fermoy, P61 C996 Co. Cork Ireland; 7 School of Food and Nutritional Sciences, University College Cork, Cork, T12 Y337 Co. Cork Ireland; 8 School of Biological, Earth and Environmental Sciences, University College Cork, Cork, T23 N73K Co. Cork Ireland; 9 Teagasc, Environmental Research Centre, Johnstown Castle, Y35 Y521 Co. Wexford Ireland

**Keywords:** Dietary regime, Metabolomic profile, Triglycerides, Pasture, Whole milk powder, Buttermilk

## Abstract

**Introduction:**

Bovine milk contains a rich matrix of nutrients such as carbohydrates, fat, protein and various vitamins and minerals, the composition of which is altered by factors including dietary regime.

**Objectives:**

The objective of this research was to investigate the impact of dietary regime on the metabolite composition of bovine whole milk powder and buttermilk.

**Methods:**

Bovine whole milk powder and buttermilk samples were obtained from spring-calving cows, consuming one of three diets. Group 1 grazed outdoors on perennial ryegrass which was supplemented with 5% concentrates; group 2 were maintained indoors and consumed a total mixed ration diet; and group 3 consumed a partial mixed ration diet consisting of perennial ryegrass during the day and total mixed ration maintained indoors at night.

**Results:**

Metabolomic analysis of the whole milk powder (N = 27) and buttermilk (N = 29) samples was preformed using liquid chromatography-tandem mass spectrometry, with 504 and 134 metabolites identified in the samples respectively. In whole milk powder samples, a total of 174 metabolites from various compound classes were significantly different across dietary regimes (FDR adjusted p-value ≤ 0.05), including triglycerides, of which 66% had their highest levels in pasture-fed samples. Triglycerides with highest levels in pasture-fed samples were predominantly polyunsaturated with high total carbon number. Regarding buttermilk samples, metabolites significantly different across dietary regimes included phospholipids, sphingomyelins and an acylcarnitine.

**Conclusion:**

In conclusion the results reveal a significant impact of a pasture-fed dietary regime on the metabolite composition of bovine dairy products, with a particular impact on lipid compound classes.

## Introduction

Milk and dairy products provide important nutrition to over 80% of the world’s population, with dairy product consumption averaging 87 kg per person yearly (Britt et al., 2018; IDF, 2023). These products contain a complex array of macro and micronutrients, the composition of which is altered through factors such as lactation stage, breed, parity and dietary regime (Haug et al., 2007; Suh, 2022). Dairy farming practises are largely influenced by region, with pasture-based feeding regimes commonly used in countries such as Ireland and New Zealand due to moderate and temperate climates with plentiful rainfall conducive to sustainable production of grass, while total mixed ration (TMR) feeding regimes are more predominant in countries such as the United States and China (Donovan et al., 2000; Elgersma et al., 2006; Gulati et al., 2018; Joubran et al., 2021). TMR dietary regimes typically consist of of conserved forages (e.g. grass, maize and corn silages), concentrates and various carbohydrates, providing a consistent diet to cows and offering farmers greater control over bovine nutrition (Arnott et al., 2017; Charlton et al., 2011; McAuliffe et al., 2018). In recent years consumer preference has grown for pasture-fed dairy products, due to the perception of enhanced health benefits and positive association with animal welfare (Conner & Oppenheim, 2008; Magan et al., 2019a; O’Neill et al., 2011; Park, 2018; Verkerk, 2003).

Dietary regime is a well-established factor influencing the chemical composition of bovine milk. Pasture-fed milk contains higher concentrations of total solids, protein, casein and fat compared to TMR-derived milks (Alothman et al., 2019; McAuliffe et al., 2018; O’Callaghan et al., 2016). The lower fat content of TMR-based milks reflects the high milk yields and the high starch content in TMR feeds and the capacity of the ruminant small intestine for digestion, influencing fat synthesis within the mammary gland (Auldist et al., 2016; Reynolds, 2006). Increasing the proportion of fresh pasture in the cow’s diet improves the nutritional value of the milk fat due to increased levels of unsaturated fatty acids (Chilliard et al., 2001; Couvreur et al., 2006). Apart from macronutrients, pasture-fed milk contains higher concentrations of micronutrients such as calcium and phosphorus (Guinee et al., 2006; Gulati et al., 2018; Tsioulpas et al., 2007). Dairy foods such as buttermilk are by-products, collected from the side-streams of butter manufacturing from cows’ milk and is widely used within the food industry (Conway et al., 2014; Lambert et al., 2016; Rose et al., 2023; Sodini et al., 2006). Buttermilk is produced from the churning of cream resulting in two phases, an aqueous phase (buttermilk) and solid phase (butter fat) (Calvo et al., 2020; Morin et al., 2007).Previous research describes how pasture-based regimes not only alter the composition milk, but downstream dairy products such as butter (O’Callaghan et al., 2016). Although the impact of pasture feeding on gross milk and dairy product composition is established there is a paucity of information in relation to the wider composition of milk and in particular using emerging technologies such as metabolomics.

Metabolomics, a technology allowing for the global characterisation of biological fluids has become a major tool within food research (Capozzi & Bordoni, 2013; Wishart, 2008). Various applications of metabolomics have identified metabolites from compound classes such as amino acids, carbohydrates, lipids, vitamins and energy metabolites in bovine milk (Boudonck et al., 2009; Foroutan et al., 2019; Rocchetti et al., 2022; Sharma & Ozogul, 2023). Previous studies have highlighted that diet is a key factor altering the levels of metabolites in bovine milk and milk products. The metabolomic profiles of milk from pasture based systems were reported to be different to samples obtained from TMR diets (O’Callaghan et al., 2018) (Magan et al., 2019a). Examination of bulk milk samples from dietary regimes such as corn silage, hay and fresh forage revealed an impact on triglycerides, glycerophospholipids and polyphenols (Rocchetti et al., 2020). In support of this, the metabolomic profile of milk obtained from cows fed a hay-or silage-based diet were distinctly different, with alterations occurring in the triglyceride profile (Wölk et al., 2022). Metabolite classes such as triglycerides influence both physical and functional properties of high fat dairy products such as butter, therefore understanding differences in their composition is of key importance to the agri-food industry (Hawke & Taylor, 1983; Ortiz Gonzalez et al., 2023). Milks from TMR based feeding contain higher levels of saturated fatty acids, attributed to higher dietary starch consumption and upregulation of de novo synthesis of fatty acids (Rodríguez-Bermúdez et al., 2023). Pasture-based milks contain higher levels of medium chained fatty acids and omega-3 polyunsaturated fatty acids compared to TMR-based milks. Numerous studies have demonstrated the beneficial alterations occurring in the fatty acid profile of milk as a result of pasture feeding (Chilliard et al., 2007; Couvreur et al., 2006; Dewhurst et al., 2006; O’Callaghan et al., 2016; Timlin et al., 2023). Maximising grass intake in Holstien-Friesian cows led to an increase in oleic, linoleic, linolenic, and conjugated linoleic acids, reducing atherogenic and thrombogenic indices of milk (Techeira et al., 2023). Collectively, these studies highlight the potential of metabolomics to investigate alterations in milk coming from animals fed different diets.

Understanding the specific modulations occurring in the metabolomic profile of milk and dairy products will strengthen the knowledge behind the potential benefits of a pasture-based regime. Therefore, the objective of this research is to characterise the metabolomic profile of bovine whole milk powder and buttermilk from pasture, TMR and partial mixed ration (PMR) dietary regimes.

## Materials and methods

### Experimental design

The feeding trial was designed to examine compositional differences between high, medium, and no pasture derived diets across a full lactation. Detailed feeding trial study information, including the composition of each dietary regime was previously described (Fitzpatrick et al., 2022; Timlin et al., 2023).

In brief, fifty-four spring-calving cows were allocated into 3 dietary regime groups (N = 18) across an entire lactation at the Teagasc, Animal and Grassland Research and Innovation Centre, Moorepark, Fermoy, Co. Cork, Ireland. The cows were predominantly Holstein Friesian, Friesian and a small proportion (< 10%) were Friesian Jersey. Cows were allocated randomly to a dietary group, based on mean calving date, pre-experimental daily milk yield, milk solids yield, economic breeding index (EBI) and lactation number. For the entire lactation the animals remained in their assigned groups. Dietary regime group 1 (pasture) cows were maintained outdoors, grazing perennial ryegrass pasture through rotational grazing (*Lolium perenne L*.), at 95% of total dry matter intake with concentrates supplemented into diet during milking for the remaining 5% of annual dry matter intake. Dietary regime group 2 (TMR) were housed indoors consuming a total mixed ration (TMR) concentrates-based diet. The TMR diet consisted of maize silage, grass silage and concentrates at a ratio of 40:20:40 on a dry matter basis. Dietary regime group 3 consisted of a combination of group 1 and group 2 diets. Dietary group 3 (PMR) consisted of 4.5 kg of concentrates, 4.5 kg of maize silage, 2.25 kg of grass silage and 9 kg of grass silage (Fitzpatrick et al., 2022). Furthermore, cows in group 3 (PMR) consumed a diet consisting of 95% pasture-based diet between morning (a.m.) and evening (p.m.) milkings, then consuming a TMR diet between p.m. and a.m. milkings. The feeding trial was not blinded.

### Whole milk powder sample manufacture

Whole milk powders (WMP) (N = 27) were manufactured in Moorepark Technology Limited (MTL; Fermoy, Co. Cork, Ireland), during early (March 2021), mid (July 2021) and late (October 2021) lactation. The milk from each herd fed pasture, TMR or PMR diets was pumped into individual stainless steel agitated tanks and standardised. Milk samples were chilled overnight and pasteurised using an APV Fisher stainless steel pasteuriser (SPX Flow Technology Denmark A/S, Soeborg, Denmark) with the resulting pasteurised milks homogenised using a two-stage homogeniser at 8000 kPA and 2000 kPA (Model NS2006H, Niro Soavi, Parma, Italy). The homogenised milks were evaporated to 45% solids (Anhydro F1 Lab, Copenhagen, Denmark), with spray drying conducted using a single stage dryer (Anhydro Spray Dryer, SPX Flow Technology Denmark A/S, Soeborg, Denmark). All samples collected were stored in airtight containers, wrapped in aluminium foil and kept at room temperature until analysis.

### Buttermilk sample manufacture

Milk was collected for buttermilk manufacturing trials in early (March 2020, April 2021), mid (July 2020) and late (October 2020) lactation. Within the early lactation sample set, two samples were produced in March 2020 however to the national COVID-19 lockdown production was suspended, with the remaining samples produced in April 2021. Milk from each dietary group was collected and transported to Moorepark Technology Limited (MTL, Moorepark, Fermoy, Co. Cork, Ireland) for manufacturing. Milk was pasteurized using a Unison pasteurizer (Unison Engineering Ltd., Limerick, Ireland) at 72 °C for 15 s. Following this the cream (38–40% fat) was separated using a centrifugal disk (Westfalia separator d-4740, GEA, Naas, Ireland) at 50 °C and cooled rapidly by recirculation through a plate heat exchanger. The cream was stored overnight at 4 °C. The butter churn (A·S·T·A eismann GmbH, Neubeckum, Germany) was washed with hot water, stored in a diluted Super-Sil detergent solution (Biocel Ltd., Little Island, Co. Cork, Ireland) and held overnight before rinsing with chilled reverse osmosis treated water immediately prior to cream processing. Cream (20–40 kg) was added to the butter churn and churned until the solid and liquid phases inverted. Buttermilk (liquid phase) was drained and samples were frozen at − 20 °C until further analysis.

### Gross compositional analysis

Analysis of components including fat, protein, lactose, total nitrogen, moisture and ash was preformed on whole milk powder samples (N = 27). Total nitrogen and protein content were quantified using the Kjeldahl method. Detailed information on the general milk composition analysis is previously described (Timlin et al., 2023).

## Metabolomic analysis

### LC–MS/MS whole milk powder sample preparation

Reconstituted whole milk powder samples were assembled for targeted metabolomic analysis according to the MxP® Quant 500 assay manual (Biocrates Life Sciences, Innsbruck, Austria) (Connolly et al., 2023). The MxP® Quant 500 assay allows for the identification of metabolites from various metabolite classes, of which 13% include small molecules from compound classes such as amino acids and biogenic amines. Reconstitution of the whole milk powder was achieved by adding 10 mL of 37 °C High Performance Liquid Chromatography (HPLC) H_2_O to 1 g of whole milk powder, with resulting milk samples shaken at room temperature for an hour. The samples were randomised, and 10 μL was added to the filter inserts of the 96 well plate and dried under a continuous nitrogen stream at room temperature for 30 min. Once dried, derivatization solution (50 μL) was added to the plate and incubated for 25 min, then dried under a nitrogen stream for 60 min. Metabolites were then extracted (19 mg ammonium acetate in 50 ml HPLC grade methanol) and centrifuged at 500 g for 2 min. For LC–MS/MS analysis, 150 μL of the resulting eluate was combined with HPLC grade H_2_O (150 μL). Furthermore, to preform flow injection analysis tandem mass spectrometry (FIA-MS/MS), 50 μL eluate was added to the running solvent. Analysis was conducted using a Sciex ExionLC series UHPLC system coupled to a Sciex QTRAP 6500 + mass spectrometer. The UHPLC column provided by Biocrates Life Sciences (Biocrates Life Sciences, Innsbruck, Austria) was employed and 100% water and 95% acetonitrile (both added 0.2% formic acid) were prepared as mobile phase A and B, respectively. Compounds including amino acids and related compounds, bile acids, biogenic amines, carboxylic acids and some other metabolites such as trigonelline, trimethylamine N-oxide and choline were also quantified in positive and negative mode using LC–MS/MS. Lipid classes including lysophosphatidylcholines, phosphatidylcholines, sphingomyelins, ceramides, cholesteryl esters, diglycerides and triglycerides, acylcarnitines and the sum of the hexose were semi-quantified in positive mode FIA-MS/MS analysis. Metabolites were identified and quantified using the multiple reaction monitoring (MRM) method.

### LC–MS/MS buttermilk sample preparation

Buttermilk samples were defrosted at room temperature for an hour, then prepared and measured according to the AbsoluteIDQ p180 assay manual (Biocrates Life Sciences, Innsbruck, Austria). Sample preparation was similar to the whole milk powder outlined above with 10 μL of buttermilk samples used in preparation of the 96 well plate (Connolly et al., 2023). Data was acquired on a Sciex QTRAP 6500 + mass spectrometer coupled to a Sciex ExionLC series UHPLC capability as previously described. Data acquisition was performed using AB Sciex Analyst software. Amino acids (N = 16) and biogenic amines (N = 12) were quantified in positive mode. Using FIA-MS/MS analyses, 13 acylcarnitines, 10 lysophosphatidylcholines, 68 phosphatidylcholines, 14 sphingomyelins, and the sum of hexoses (H1) were identified and quantified in positive mode. These metabolites were measured semi-quantitatively by using 14 internal standards.

### Data processing and metabolites quantification

In both sample sets, metabolite levels are reported in micromoles and data quality was evaluated by investigating the accuracy and reproducibility of the quality control sample, provided with both the Quant 500 and P180 assays. The data quality was evaluated within the MetIDQ software (Biocrates Life Sciences AG, Austria), and metabolites were included for further statistical analyses only if their levels were above the limit of detection (LOD) in > 75% of buttermilk or wholemilk powder samples.

### Statistical analysis

Data analysis was performed using MetaboAnalyst 5.0 (www.metaboanalyst.ca). Prior to statistical analysis the datasets were normalized to the total sum of each sample. Data filtering in the form of interquartile range (IQR) filtering (25%) was applied to the whole milk powder dataset. General linear model (GLM) analysis was applied to both datasets to assess the impact of dietary regime on the metabolite composition controlling for lactation stage as a covariate. All p-values were adjusted for multiple comparisons using the Benjamini and Hochberg false discovery rate (FDR) procedure, with FDR ≤ 0.05 considered statistically significant. Posthoc analysis was performed with the Tukey test to identify where the groups that were significantly different. Graphs were generated using R (Version 4.1.2 (circus plot: circlize)).

## Results

### Gross compositional analysis of whole milk powder

The macronutrient composition of the whole milk powder was relatively stable across the three dietary regimes (Table 1). There were no significant differences in macronutrients across the three feeding regimes.

**Table 1:** Gross composition of whole milk powder samples from each dietary regime (N = 27)

	Dietary regime			
	Pasture (N = 9)	PMR (N = 9)	TMR (N = 9)	P-*value*
Fat (g/100 g)	28.66 ± 1.20	27.06 ± 0.61	27.80 ± 0.62	0.548
Protein (g/100 g)	27.81 ± 0.65	27.11 ± 0.39	26.80 ± 0.60	0.881
Total Nitrogen (g/100 g)	4.36 ± 0.10	4.25 ± 0.06	4.20 ± 0.09	0.881
Moisture (g/100 g)	1.85 ± 0.72	1.76 ± 0.68	1.79 ± 0.70	0.986
Ash (g/100 g)	5.74 ± 0.10	5.91 ± 0.23	5.87 ± 1.27	0.486
Lactose (g/100 g)	35.93 ± 1.60	38.16 ± 0.79	37.74 ± 0.70	0.419

Data is presented as mean (SD) of the relative concentration (g/100 g) for milk components for each dietary regime; Pasture (N = 9), PMR (N = 9) and TMR (N = 9). General linear model analysis controlling for lactation stage was used to determine the significant components across dietary regimes, with an FDR adjusted p-value ≤ 0.05 considered statistically significant. Abbreviations are as follows. N; number of samples, SD; standard deviation, PMR; Partial mixed ration, TMR; Total mixed ration, FDR; false discovery rate

### Whole milk powder from pasture-fed cows had a significantly altered metabolomic profile

**Fig. 1:**
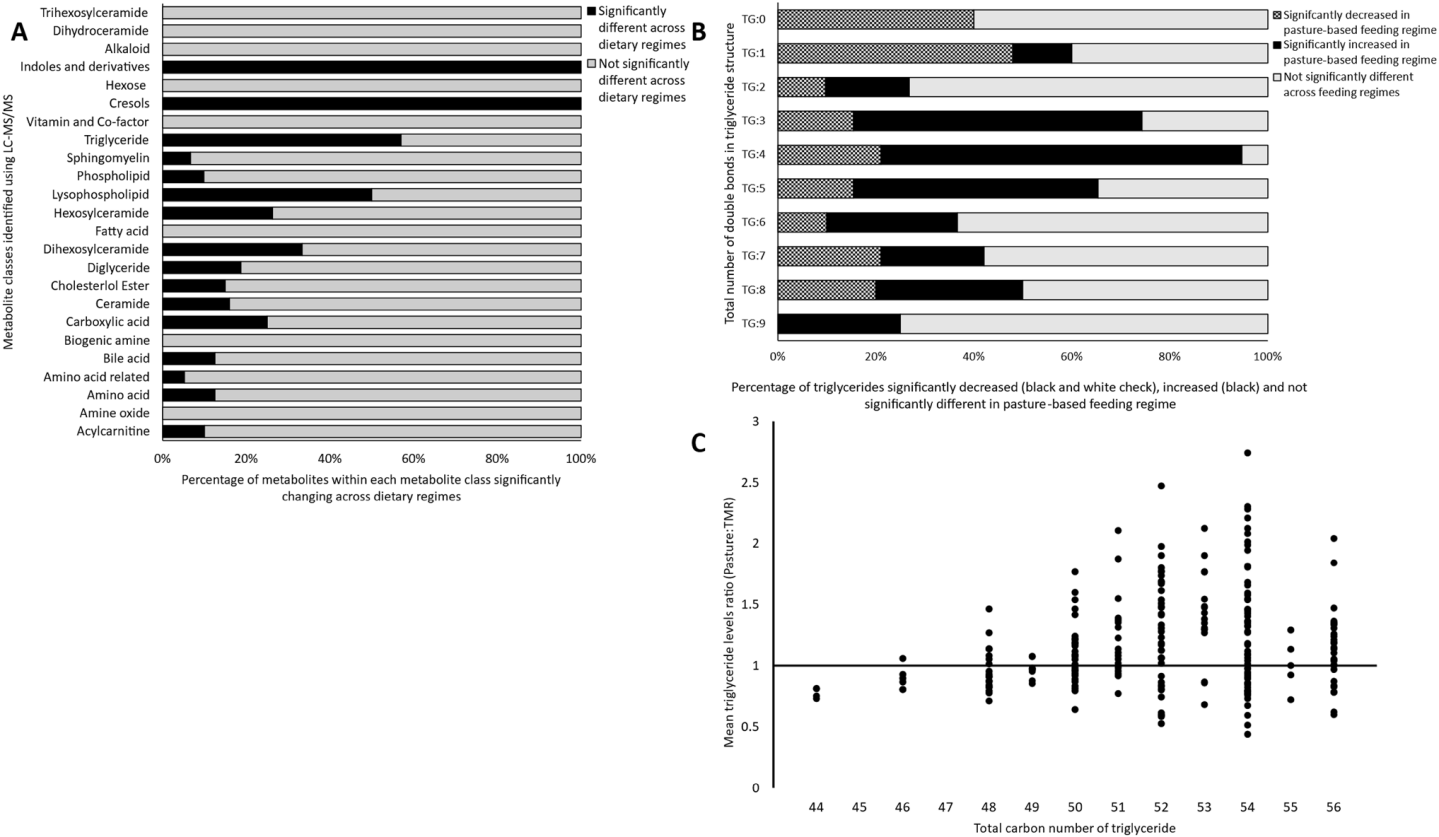
Alterations occurring in individual metabolite classes as a result of dietary regime and the modifications of the triglyceride profile in whole milk powder. **A** Stacked bar chart depicting the metabolite classes targeted by LC–MS/MS analysis of bovine whole milk powder data across three dietary regimes; Pasture, PMR and TMR (N = 27). General linear model analysis controlling for lactation stage was used to determine statistical differences, with an FDR adjusted p-value ≤ 0.05 considered statistically significant. **B** Stacked bar chart based on the total number of double bonds in triglycerides, depicting the significantly decreased in pasture-feeding (black and white check), increased in pasture-feeding (black) and not significantly changing (grey). **C** The mean ratio of triglyceride levels in pasture versus TMR feeding regimes based on the total carbon number of the triglyceride, in whole milk powder samples (N = 27). Abbreviations are as follows. *TMR* total mixed ration, *PMR* partial mixed ration, *FDR* false discovery rate, *LC–MS/MS* liquid chromatography tandem mass spectrometry

**Fig. 2:**
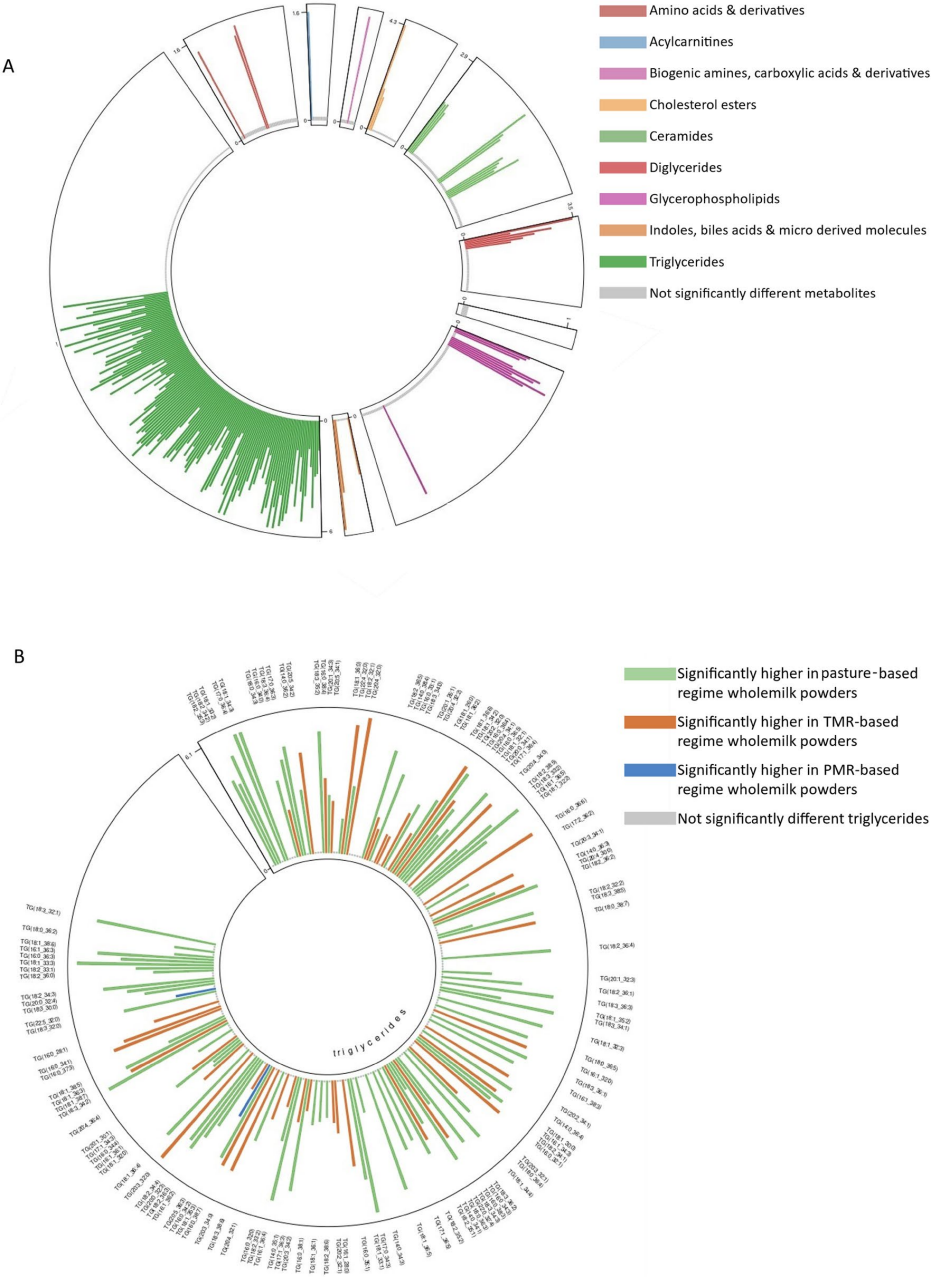
The impact of dietary regime on metabolite classes of whole milk powder. **A** Circos plot showing the significantly different metabolites across three dietary regimes; Pasture, PMR and TMR (N = 27). General linear model analysis controlling for lactation stage was used to determine the significant metabolites with an FDR adjusted p-value ≤ 0.05 considered statistically significant. The coloured bars represent significant metabolites (FDR adjusted p-value ≤ 0.05), coloured by metabolite compound class. Where no bar is present that metabolite was not significantly different. Abbreviations are as follows. **B** Circos plot showing the significantly different triglyceride metabolites (FDR adjusted p-value ≤ 0.05). The coloured bars represent significant metabolites coloured by dietary regime in which the highest metabolite level is observed pasture (green), PMR (blue) and TMR (orange) (N = 27). Abbreviations are as follows. *TG* triglyceride, *TMR* total mixed ration, *PMR* partial mixed ration, *FDR* false discovery rate

**Fig. 3:**
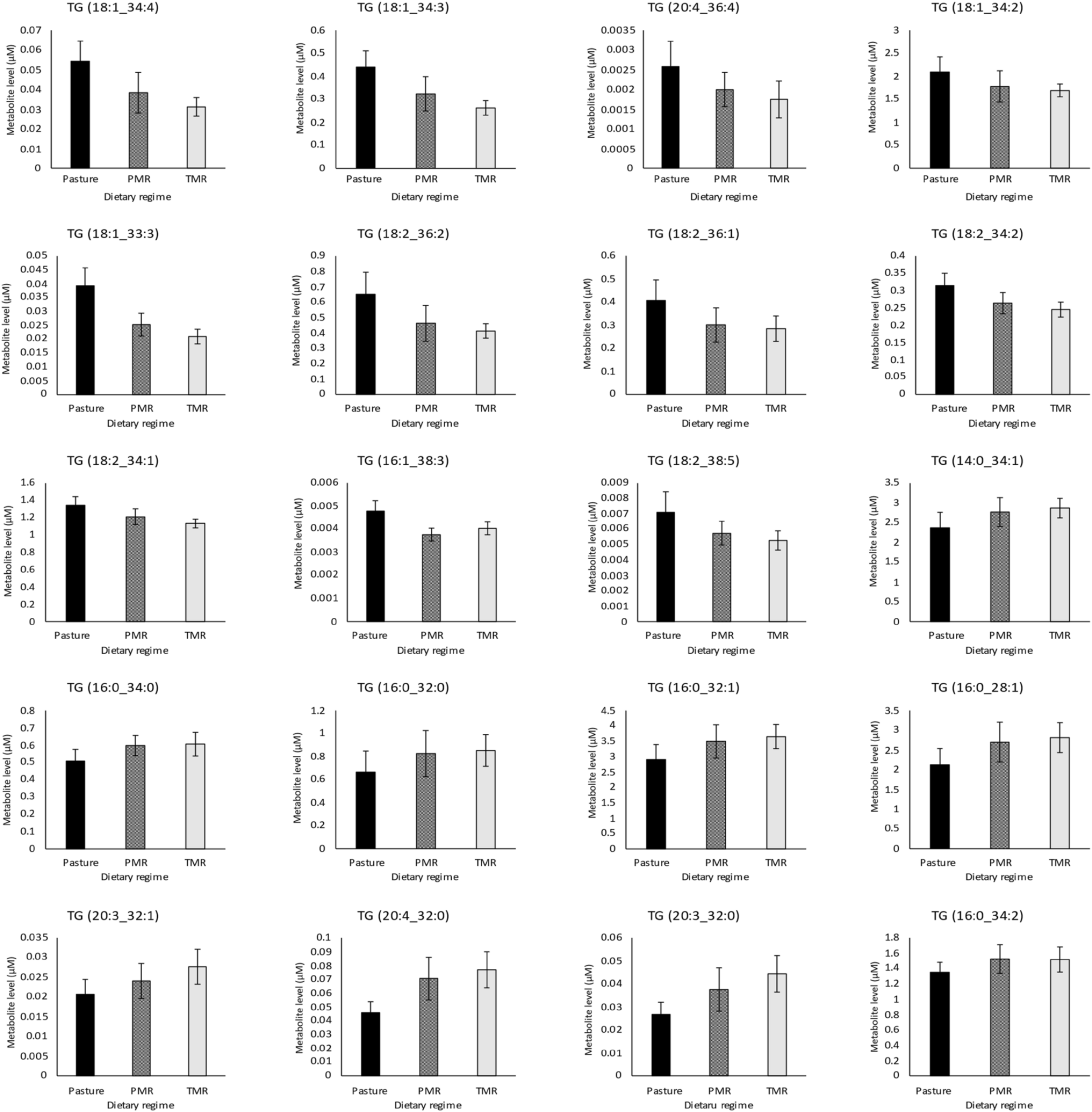
Bar graphs of significantly different triglyceride metabolites across three dietary regimes in wholemilk powder samples: Bar graphs of significantly different triglycerides across three dietary regimes; Pasture, PMR and TMR in wholemilk powder samples (N = 27). General linear model analysis controlling for lactation stage was used to determine the statistical differences with an FDR adjusted p-value ≤ 0.05 considered statistically significant. Bar graphs are coloured according to dietary regime; pasture (black), PMR (black and white check) and TMR (light grey). Data is presented as mean levels for each metabolite, error bars represent the standard deviation for each metabolite. Abbreviations are as follows. *PMR* partial mixed ration, *TMR* total mixed ration; *FDR* false discover rate

Using LC–MS/MS, a total of 504 metabolites were identified in whole milk powder samples representing metabolites from 24 different classes (Fig. 1A). Detailed analysis of the metabolomic data revealed that 174 metabolites were significantly different across dietary regimes, affecting 16 different metabolite classes (FDR adjusted p-value ≤ 0.05) (Fig. 1A). Metabolites with the highest levels among measured compounds in the milk samples included triglycerides (TG) such as TG (18:1_34:1), TG (18:1_32:1), TG (18:1_30:0), TG (18:1_32:0) and TG (16:0_34:1). Two amino acids, histidine and threonine had significantly lower levels in pasture derived whole milk powders. Additionally, 83% (5/6) of significant diglycerides had their highest levels in pasture-based milk powders, all of which had an oleic acid (C18:1) as one fatty acid within the diglyceride structure. A total of 6 phospholipids were significantly different, all of which had their lowest levels in pasture-derived milk powders compared to TMR-based powders. The significantly different phospholipids had varying acyl chain lengths from C30-38, of which two were saturated and four were polyunsaturated. Similarly, lipid classes such as ceramides, hexosylceramides and dihexosylceramides had significantly lower levels in pasture-derived milk powders. The triglyceride compound class was affected by dietary regime, with 57% (135/237) of the total triglycerides analysed different across dietary regimes (Fig. 2A). Analysis of the triglycerides found that 66% (90/135) of significant triglycerides had their highest levels in pasture-derived milk powders (Fig. 3), with a large proportion of these triglycerides containing polyunsaturated fatty acid side chains (Fig. 1B, C, Fig. 3). Additionally, pasture-derived milk powders contained significantly decreased levels of triglycerides containing saturated fatty acid side chains which were observed as significantly higher in TMR-based milk powders (Fig. 1B, C, Fig. 3). Analysis of the triglycerides containing three, four or five double bonds (polyunsaturated triglycerides) demonstrated that 65% (64/98) of these were found in significantly higher levels in pasture-derived milk powders (Fig. 1B). Additionally, 51% (60/118) of the triglycerides that were significantly higher in pasture-derived powders had a high total carbon number (carbon number 52–54) (Fig. 1C).

### Alteration in lipid metabolite classes in pasture-fed buttermilk samples

**Fig. 4:**
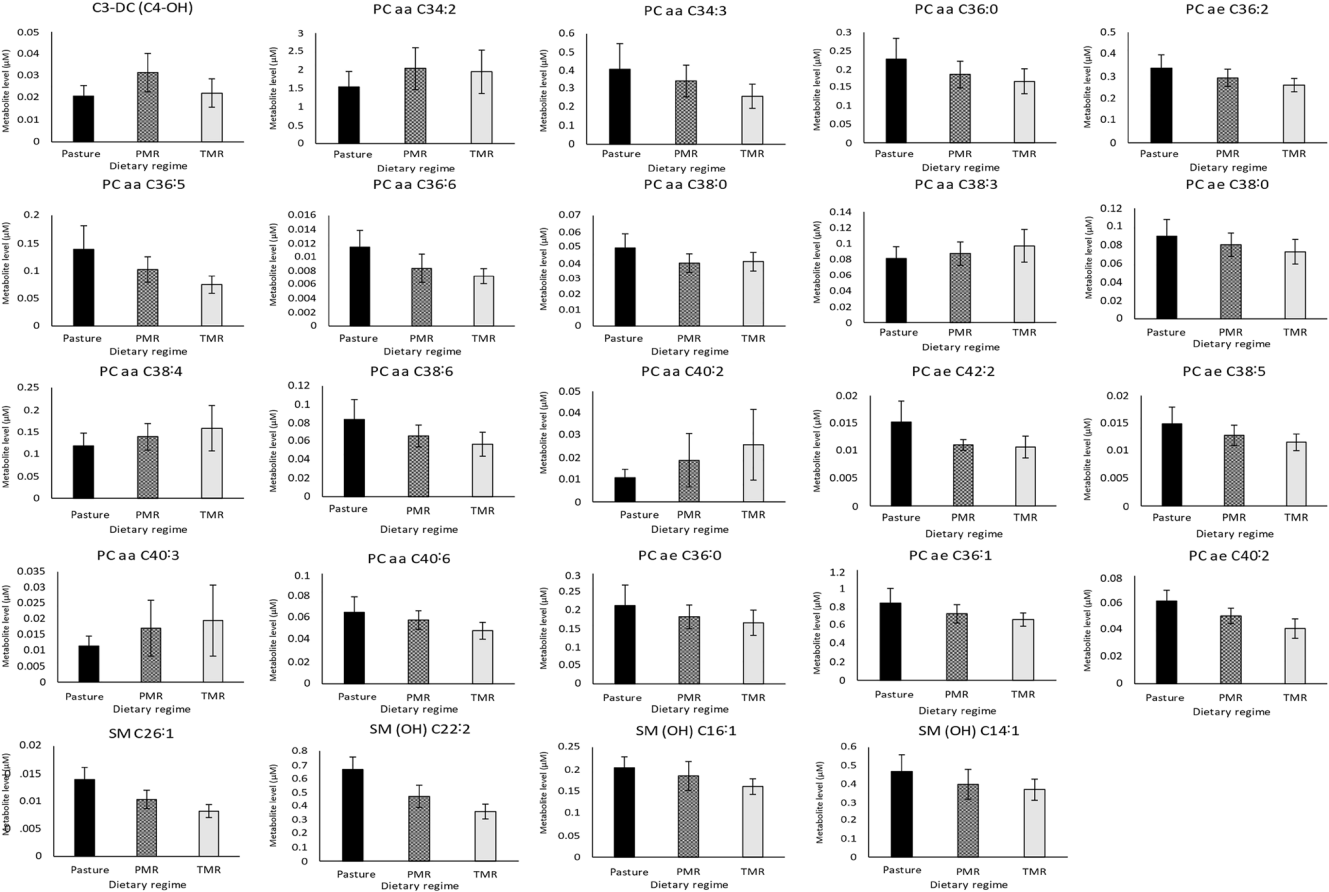
A pasture-fed dietary regime alters lipid metabolites of bovine buttermilk: Bar graphs of metabolites significantly different across three dietary regimes; Pasture, PMR and TMR (N = 29). General linear model analysis controlling for the impact of lactation stage was used to determine the percentage of statistically significant metabolites changing across dietary regimes, with an FDR adjusted p-value ≤ 0.05 considered statistically significant. Bar graphs are coloured according to dietary regime; pasture (black), PMR (black and white check) and TMR (light grey). Data is presented as mean levels for each metabolite, error bars represent the standard deviation for each metabolite. Abbreviations are as follows. *PMR* partial mixed ration, *TMR* total mixed ration, *FDR* false discover rate

In the buttermilk samples, 134 metabolites were identified from 7 metabolite classes including acylcarnitines (N = 13), amino acids (N = 16), biogenic amines (N = 12), lysophospholipids (N = 10), phospholipids (N = 68), sphingomyelins (N = 10) and one hexose (N = 1). Metabolites with highest levels among measured compounds within buttermilk samples included hexoses (H1), glutamate, glycine, creatinine and PC aa C34:1. Similar to the whole milk powder, analysis of the buttermilk samples highlighted differences in lipid compound classes as a result of dietary regime. Metabolites significantly different across dietary regime included 19 phospholipids, 4 sphingomyelins and 1 acylcarnitine (FDR adjusted p-value ≤ 0.05) (Fig. 4). The significantly different phospholipids were predominantly those with long acyl chain lengths (chain lengths from 34 to 42 carbons) of which 4 were saturated, 1 monounsaturated and 14 polyunsaturated. For the significantly different phospholipids 74% had highest levels in pasture-fed whole milk powders (Fig. 4). Moreover, 100% of the significantly different sphingomyelins had their highest levels in pasture-fed buttermilk samples (Fig. 4).

## Discussion

Several studies highlight factors altering milk and dairy product composition, with dietary regime playing a pivotal role. Our results demonstrate a distinct metabolomic signature associated with pasture-fed whole milk powder and buttermilk, with a profound effect on lipid metabolite classes. Understanding the impact of feeding regime on bovine milk and associated dairy products is of key importance to the agri-food sector, due to its effect on downstream dairy production and consumption by the human population.

Our results indicate a clear distinction between the metabolomic profiles of pasture- versus TMR-based whole milk powders. In this study, pasture based and levels thereof had a distinct impact on metabolite classes including triglycerides, ceramides, and amino acids. Milk fat accounts for 3–5% of overall bovine milk gross composition and plays a vital role as an indicator of quality of dairy products, combining a diverse array of lipid classes including triglycerides, sphingolipids, glycerophospholipids and fatty acids (Haug et al., 2007; Jensen, 2002; Wang et al., 2022). It is well documented that pasture-fed milk has higher gross fat content, however compositional analysis of wholemilk powder samples in this study did not show any significant difference in fat content between dietary regimes. This result was in line with previous analysis of wholemilk powders, attributed to the processing, homogenisation and standardisation processes involved in the formation of powdered milk, reducing the influence of diet on the milk powder (Magan et al., 2019b, 2021). However in line with our results, previous metabolomics analysis demonstrated how prompt alterations occur in the milk lipidome and compound classes such as fatty acids, triglycerides and glycerophospholipids as a result of feeding changes (Haug et al., 2007; Liu et al., 2020; O’Callaghan et al., 2016; Rocchetti et al., 2020; Wölk et al., 2022). Our study highlighted that 135 triglycerides were significantly different across dietary regimes. The pasture-based milk powders contained significantly higher levels of triglycerides having a high total carbon number (CN54) and containing polyunsaturated fatty acid side chains, whereas TMR-derived milks contained higher levels of triglycerides containing lower carbon numbers. In accordance with these results, a study which investigated the impact of pasture feeding on the bovine milk triglyceride profile reported that milks produced in spring-early summer contained higher levels of triglycerides with a carbon number of 54, attributed to the uptake in fresh-grass within the diet (Capuano et al., 2014). In support of this, a study which investigated the impact of hay versus silage dietary regimes found that triglycerides with a carbon number of 50 or greater were increased in the summer period milk as a result of increased fresh pasture consumption (Wölk et al., 2022). Many studies corroborate these findings that pasture-based milks contain a higher quantity of triglycerides with higher total carbon number, attributed to the presence of α-linoleic acid (C18:1 fatty acid) within grass (Balcazar et al., 2022; Capuano et al., 2014; Gresti et al., 1993; Tzompa-Sosa et al., 2016). Pasture-fed milk contains significantly higher levels of polyunsaturated triglycerides and omega-3 fatty acids resulting in a preferential thrombogenic ratio, which offers enhanced nutritional value, thus highlighting the superior nutritional qualities of pasture-fed milk and associated dairy products in human nutrition (Abedi & Sahari, 2014; Capuano et al., 2014; Haug et al., 2007; Heins, 2021; Lorgeril et al., 1994).

The present study reports that triglycerides containing saturated fatty acids were higher in TMR-derived milks. Previous studies have shown that a diet consisting of silage and concentrates in colder months resulted in the production of milk with higher levels of saturated triglycerides when compared to milk produced from cows fed fresh fodder during summer months (Wölk et al., 2022). The degree of unsaturation of triglycerides influences the spread-ability and melting point butter, demonstrating the importance of milk lipid composition in downstream dairy product processing and highlighting how diet and in particular a pasture-fed regime may be preferential (Liu et al., 2017).While this study identified changes in the fatty acid side chains of triglyceride and phospholipids, no differences in free fatty acid levels across the different dietary regimes were observed. However, it is important to note that a limited number of free fatty acids were measured and they were all even chain fatty acids. Previous studies have reported an impact of pasture feeding on free fatty acids in raw milk (O’Callaghan et al., 2016; Șanta et al., 2022; Timlin et al., 2023). Furthermore, in the present study pasture-fed milk powders contained decreased levels of certain ceramides. A recent study examining the impact of starch content on milk lipids found high starch diets reduced ceramide levels in milk (Rico et al., 2021). While ceramides represent a small proportion of the total lipids in milk, their composition appears to be highly susceptible to alteration as a result of dietary change (Liu et al., 2018). Further work is warranted to fully understand the factors controlling ceramide and fatty acids levels in wholemilk powder.

It is well documented that altering dietary regime impacts not only the composition of milk produced but also products produced from the milk itself (Conway et al., 2014; Lopez, 2011; Lopez et al., 2008; Magan et al., 2019a). In our analysis, phospholipids significantly affected by dietary regime were predominantly higher in the pasture-fed buttermilk samples, of which the majority had longer acyl chain lengths and were unsaturated. In agreement with our findings, a previous study reported that buttermilk produced from cows on a pasture-based diet contained a significantly higher amount of both total lipids and phospholipids (Lopez et al., 2017). Moreover, the buttermilk obtained from pasture-fed cows contained a lower amount of saturated fatty acids (Lopez et al., 2017). Buttermilk is a rich source of polar lipids such as phospholipids and sphingomyelins, due to their high content in the milk fat globule membrane (Dewettinck et al., 2008; Gassi et al., 2016; Lambert et al., 2016; Rombaut et al., 2006). The milk fat globule membrane is a membrane structure which is found in the aqueous phase of milk containing various lipid classes, which becomes damaged during butter production resulting in fragments being released into the buttermilk produced, leading to high quantities of membrane within buttermilk (Gassi et al., 2016; Rombaut et al., 2006). In our study, significant sphingomyelins had their highest levels in pasture-fed buttermilk samples. Bioactive compounds such as sphingomyelins found in milk, are a main source of sphingolipids within the human diet, the composition of which are altered by diet (Gassi et al., 2016; Lopez et al., 2008, 2017). Altogether, our results highlight the positive impact of a pasture-based regime on phospholipid and sphingomyelin levels in buttermilk samples, demonstrating the potential enhancement in the nutritional profile in pasture-based buttermilk products.

In summary, our results demonstrate significant impact of a pasture-based feeding regime on the metabolomic profile of dairy products such as whole milk powder and buttermilk, when compared to TMR or PMR dietary regimes. The results of this study contribute to the current knowledge of how diet impacts milk composition and in particular demonstrated key metabolite differences within lipid compound classes. However, further research is warranted to understand the effect of diet on the animals metabolomic profile. The present analysis provides additional insight into the potential positive health benefits of consumption of pasture-fed milk and dairy products.

## Conclusion

Our study examined the metabolomes of whole milk powder and buttermilk across three different dietary regimes, attempting to better understand complex mechanisms linking dietary regime and milk composition. In total, 504 and 134 metabolites were identified and quantified in whole milk powder and buttermilk samples respectively with distinctive differences in lipid compound classes in both sample types. In whole milk powder samples, dietary differences between pasture- and TMR-based samples were driven by alterations in the triglyceride composition of the milk powders. Strengths associated with this analysis include its targeted approach, providing semiquantitative results of compound classes including amino acids, biogenic amines, phospholipids and triglycerides. Our observed differences corroborate with the literature, however future studies should include analysis of other dairy products in order to allow precise conclusions on this impact in downstream dairy product production. Furthermore, this analysis should be conducted using a multi-platform metabolomic approach including both nuclear magnetic resonance spectroscopy and gas chromatography to provide extensive metabolite coverage. Finally, future studies should also evaluate whether consumption of products from different farming regimes influences the human metabolomic profile.

## Data Availability

The metabolomics data presented in this study are available on request from the corresponding author.

## Declarations

### Research involving human and animal participants

All applicable international, national, and/or institutional guidelines for the care and use of animals were followed.
